# Psychiatric Conditions Following Surgical Interventions: A Case Series

**DOI:** 10.1192/j.eurpsy.2025.1219

**Published:** 2025-08-26

**Authors:** Y. M. Akkaya, K. Şahin Akkaya, H. Kaya

**Affiliations:** 1 Psychiatry, Erenkoy Mental and Nervous Diseases Training and Research Hospital, Istanbul, Türkiye

## Abstract

**Introduction:**

The surgical treatment process involves not only physical recovery but also the management of psychiatric and psychosocial issues. Psychiatric disorders can negatively affect postoperative recovery, complicate adherence to treatment and decrease the quality of life (Begum *et al.* World J Surg 2022; 46(6) 1408-1419).

**Objectives:**

This case series highlights less commonly encountered psychiatric conditions that arise after surgery and emphasizes the importance of considering how these conditions interact with pre-existing diseases during postoperative follow-up.

**Methods:**

This case series examines three distinct cases of psychiatric disorders following surgical interventions:

Delusional disorder after hypophysectomy

Somatization disorder after cystoscopy

Psychotic depression following colostomy creation

Informed consent was obtained from all patients.

**Results:**

**Case 1**

A 62-year-old male patient diagnosed with hypophyseal macroadenoma underwent transsphenoidal hypophysectomy. Two months after surgery, he developed paranoid delusions, believing his wife was having an affair. Initially treated with aripiprazole 15 mg/day, the patient did not improve. His treatment was switched to risperidone 2 mg/day, resulting in resolution of his symptoms.

**Case 2**

A 58-year-old male with benign prostatic hyperplasia (BPH) developed persistent groin pain after cystoscopy. Despite urological treatment, the pain did not subside, and he was referred to algology for gabapentin, which was ineffective. Referred to psychiatry, he reported pain radiating to his back and arms, worsened by stress, and trouble sleeping due to his pain. Diagnosed with somatization disorder, he was treated with olanzapine 2.5 mg/day and cognitive interventions, which led to decreased pain.

**Case 3**

A 72-year-old male patient with rectal cancer, following abdominoperineal resection and colostomy creation, began consuming other people’s medications. He exhibited disorganized behavior and suicidal ideation, and was diagnosed with psychotic depression. Treated with olanzapine 5 mg/day and venlafaxine 75 mg/day, his disorganized behavior resolved during follow-up, and olanzapine was discontinued. He remains in remission on venlafaxine 75 mg/day.

**Conclusions:**

This case series illustrates the diversity of psychiatric conditions that can arise after surgical interventions and emphasizes the importance of postoperative psychiatric monitoring. Although there is a lack of sufficient studies on this topic, a postoperative follow-up study conducted with a group of 200 patients found that the risk of developing anxiety after surgery was 31%, and the risk of developing depression was 56% (Basak *et al.* Int J Surg 2015; 23 18-22). Psychiatric symptoms can complicate physical recovery, affect adherence to treatment, and reduce quality of life. A multidisciplinary approach is essential to support both physical and psychological recovery, ultimately improving the overall health status of patients.

**Disclosure of Interest:**

None Declared

